# Cross-reactivities between human IgMs and the four serotypes of dengue virus as probed with artificial homodimers of domain-III from the envelope proteins

**DOI:** 10.1186/1471-2334-13-302

**Published:** 2013-07-01

**Authors:** Nora Zidane, Philippe Dussart, Laetitia Bremand, Hugues Bedouelle

**Affiliations:** 1 Department of Infection and Epidemiology, Institut Pasteur, Unit of Molecular Prevention and Therapy of Human Diseases, rue du Docteur Roux, F-75015 Paris, France; 2 CNRS URA3012, rue du Docteur Roux, F-75015 Paris, France; 3 Institut Pasteur de la Guyane, Laboratory of Virology, National Reference Center for Arboviruses, Cayenne, French Guiana

**Keywords:** Cross-reactivity, Dengue Virus, Discrimination, Flavivirus, Human Serums, Immunoglobulin M, MAC-ELISA, ROC Curve, Serotype, Specificity

## Abstract

**Background:**

Dengue fever is the most important vector-borne viral disease. Four serotypes of dengue virus, DENV1 to DENV4, coexist. Infection by one serotype elicits long-lasting immunity to that serotype but not the other three. Subsequent infection by a different serotype is a risk factor for severe dengue. Domain III (ED3) of the viral envelope protein interacts with cell receptors and contains epitopes recognized by neutralizing antibodies. We determined the serotype specificity and cross-reactivity of human IgMs directed against ED3 by using a well-characterized collection of 90 DENV-infected and 89 DENV-uninfected human serums.

**Methods:**

The recognitions between the four serotypes of ED3 and the serums were assayed with an IgM antibody-capture ELISA (MAC-ELISA) and artificial homodimeric antigens. The results were analyzed with Receiving Operator Characteristic (ROC) curves.

**Results:**

The DENV-infected serums contained IgMs that reacted with one or several ED3 serotypes. The discrimination by ED3 between serums infected by the homotypic DENV and uninfected serums varied with the serotype in the decreasing order DENV1 > DENV2 > DENV3 > DENV4. The ED3 domain of DENV1 gave the highest discrimination between DENV-infected and DENV-uninfected serums, whatever the infecting serotype, and thus behaved like a universal ED3 domain for the detection of IgMs against DENV. Some ED3 serotypes discriminated between IgMs directed against the homotypic and heterotypic DENVs. The patterns of cross-reactivities and discriminations varied with the serotype.

**Conclusions:**

The results should help better understand the IgM immune response and protection against DENV since ED3 is widely used as an antigen in diagnostic assays and an immunogen in vaccine candidates.

## Background

Dengue is a mosquito-borne infection of the tropics and subtropics. Some 2.5 billion people are at risk, and 50–100 millions are infected annually. Most infections are either asymptomatic or result in dengue fever, a relatively mild illness. However, a life threatening form, severe dengue, develops in 1–5% of infections [1].

Dengue viruses have been divided into four serotypes, differing in overall amino acid sequence by ≥30% [2]. Infection by DENV raises lifelong immunity against the infecting serotype but only transient protection against the other serotypes [3]. Subsequent infections by viruses from different DENV serotypes are associated with a greater risk for severe dengue [4]. The preferential reactivation of the memory B and T cells that correspond to a primary infection, and an antibody-dependent enhancement (ADE) of infection constitute triggering mechanisms of severe dengue during a secondary infection by a different viral serotype [5,6].

The IgMs are the first antibodies to appear after a primary DENV infection [7,8]. Murine and simian IgMs have been shown to neutralize DENV in vitro and be devoid of ADE activity [9,10]. The IgMs play a role in the immune response after a vaccination by a live attenuated DENV and a challenge by the homotypic virus in monkeys. The immunized animals exhibit an earlier increase of the IgM response than control animals and there is evidence for an anamnestic IgM response [11,12]. A similar observation has been made for monkeys vaccinated with a recombinant domain III of the viral envelope protein [13,14] (see below).

Immunochemical assays are commonly used to detect DENV-specific IgM and IgG antibodies. Because high affinity IgGs can compete with IgMs for antigen binding, especially during a secondary infection, an IgM capture assay is preferably used. In an IgM antibody-specific capture ELISA (MAC-ELISA), the virus specific IgMs in the test human serum are detected by first capturing all the serum IgMs through antibodies that are specific to human IgMs and bound to a solid phase [15]. The serotype specificity and cross-reactivity of the MAC-ELISA assays have been studied with various forms of viral antigen: extracts of suckling mouse brains (SMB) infected with DENV, culture supernatants of mammalian Vero cells or insect C6/36 cells infected with DENV; non-infectious virus-like particles (VLP). These studies have shown that the IgM response is serotype cross-reactive [15-18]. With SBM extracts, some monotypic responses may be observed but they frequently do not correlate with the virus serotype isolated from a patient [19]. With cell culture supernatants in contrast, the highest response is always obtained for the infecting serotype [16,17].

The dengue viruses are enveloped RNA viruses. The structures of the whole virus and of its envelope (E) protein have been solved by electron cryo-microscopy and X-ray crystallography, with the E protein either in a free state or in complex with an antibody [20-24]. Ninety dimers of the E protein cover the surface of the virus. Each E protein monomer comprises three ectodomains, ED1 to ED3, and a transmembrane segment. ED2 includes the dimerization interface, glycosylation sites and the peptide of fusion with the cellular membrane. ED3 is continuous and comprises residues 296–400 of the E protein (DENV1 numbering). Its fold is compact, immunoglobulin-like and stabilized by a disulfide bond between residues Cys302 and Cys333. The structures of recombinant ED3 domains have been solved by X-ray crystallography or NMR methods, either in a free state or in complex with an antibody [25-33]. The structure of the isolated ED3 domain is close to its structure in the E protein.

The ED3 domain participates in the interaction between the virus and primary or secondary cell receptors, including heparan sulfates and ribosomal protein SA [34-41]. Consistently, recombinant ED3 domains from DENV1 and DENV2 inhibit infectivity of the cognate virus [36,42-44]. Mutations in the ED3 domain of DENV2 affect its cell tropism and virulence [45]. The ED3 domain contains epitopes for neutralizing IgM antibodies [46]. IgM antibodies to ED3 in human serums constitute a large fraction of the total IgMs to the E protein in both primary and secondary immune responses. In contrast, IgG antibodies to ED3 constitute only a small fraction of the total IgGs to the E protein [47,48].

ED3 domains have been used as antigen in indirect IgM or IgG ELISA to detect infections by DENV [49-51]. Many studies have shown that the isolated ED3 domains from the four DENV serotypes are immunogenic in mice and elicit neutralizing and protective antibodies [52-56]. The ED3 domain from DENV2 (ED3.DENV2) elicits neutralizing antibodies and partial protection in monkey against the cognate virus [56]. Multivalent ED3 domains, i.e. single polypeptides including the ED3 domains from several serotypes of DENV, elicit neutralizing and protective antibodies in mice simultaneously to the corresponding DENV serotypes [57-59]. Recombinant or synthetic genes coding for a single or several ED3 domains in tandem have been inserted in the genome of infective non-pathogenic viruses and shown to elicit neutralizing antibodies in mice, e.g. using adenovirus or measle vaccine virus as vectors [60-62].

Here, given the importance of the ED3 domain for the life cycle of the virus and for diagnostic and vaccinal applications, we analyzed the cross-reactivities between the IgMs of human patients infected by any one of the four DENV serotypes and the ED3 domains from the four serotypes in MAC-ELISA assays. We used dimeric hybrids, (H6-ED3-PhoA)_2_, as antigens; they included a hexahistidine tag, an ED3 domain and an improved *E. coli* alkaline phosphatase. We assayed human serums whose infectious status had been carefully established. We analyzed the results of the MAC-ELISAs with Receiver Operating Characteristic (ROC) curves because they provide a global parameter, the accuracy of the test, that does not depend on the choice of a threshold in the test. These analyses gave statistical data on the capacity of the ED3 domain of each serotype: i) to distinguish between human serums infected by one of the DENV serotypes and uninfected serums; and ii) to distinguish between serums infected by a homotypic DENV and serums infected by a heterotypic DENV. They also gave data on the serotypes of the ED3 domain that are recognized by the IgMs of a serum infected by a given DENV serotype. The results showed that each viral serotype generated a specific pattern of specificity and cross-reactivity.

## Methods

### Reagents and buffers

PBS (phosphate buffered saline), Tween 20, 4-nitrophenyl phosphate (pNPP) and goat antibodies to human IgMs were purchased from Sigma-Aldrich; bovine serum albumin (BSA) from Roche; low-fat milk powder from Regilait; Maxisorp ELISA plates from NUNC. Buffer A contained 0.1% Tween 20 in PBS; buffer B, 5% (w/v) low-fat milk powder in buffer A; buffer C, 1% (w/v) low-fat milk powder in buffer A; buffer D, 10% diethanolamine, pH 9.8, 10 mM MgSO_4_, 20 μM ZnCl_2_.

### Bacterial, plasmid and viral strains

The plasmids encoding the H6-ED3-PhoA hybrids have been described [41]. Table 1 gives the origin of the viral ED3 domain and the corresponding segment of the envelope protein. Table 2 gives the number of residue changes between the ED3 domains of any two DENV serotypes, and also between the ED3 domain of any DENV serotype and the consensus ED3 domain (DENVc) [63,64]. The productions of the H6-ED3-PhoA hybrids in the periplasmic space of *E. coli* and their purification from periplasmic extracts through their His-tag were performed essentially as described [41]. The fractions of purification were analyzed by SDS-PAGE in reducing conditions. The purest fractions were pooled, snap-frozen and kept at −80°C. They were homogeneous at >95%.

**Table 1:** **Viral origins of the (H6-ED3-PhoA)**_**2 **_**dimers**

**Virus**	**Strain**	**Genbank No**	**E residues**
DENV1	FGA/89	AF226687	295-400
DENV2	Jamaica/N.1409	M20558	295-400
DENV3	PaH881/88	AF349753	293-398
DENV4	ThD4-0113-76	AY618949	295-400

The last column gives the residues of the viral E protein present in the H6-ED3-PhoA hybrid. The codons in the recombinant genes were synonymous but not necessarily identical with those in the original viral genomes.

**Table 2:** Number of residue changes between ED3 domains of different serotypes

**Serotype**	**DENV1**	**DENV2**	**DENV3**	**DENV4**	**DENVc**
DENV1	0	37	31	47	22
DENV2	37	0	42	41	25
DENV3	31	42	0	51	27
DENV4	47	41	51	0	32
DENVc	22	25	27	32	0

DENVc, consensus ED3 domain.

### Clinical samples

A first set of human serums (Group 1) was collected within the normal activity of the National Reference Center (NRC) for Arboviruses, Institut Pasteur de la Guyane, French Guiana. These serums were collected from patients who displayed clinical symptoms of dengue and whose infection by DENV was confirmed by laboratory methods. A second set of human serums (Group 2) was collected in the context of a clinical study (DENFRAME project) that was performed in French Guiana [65]. These serums were collected from patients who displayed clinical symptoms of dengue but were diagnosed as negative for DENV infection. In the following, we designate these serums as DENV-uninfected serums. Both Group 1 and Group 2 serums consisted of a series of blood samples collected for the first one during the viremic phase of the disease (from day 0 to day 4 after fever onset) and for a second one during the early convalescent phase (day 5 or later). The samples were characterized by standard diagnostic methods that included virus isolation on mosquito cells and/or viral RNA detection by RT-PCR, non-structural NS1 protein detection, as well as MAC-ELISA and IgG-specific indirect ELISA using virus-infected suckling mouse brain (SMB) extracts as antigens. For the serums of Group 1, the DENV serotype that was responsible for the disease, was identified by RT-PCR and the presence of IgMs against the infecting DENV serotype was ascertained from a MAC-ELISA that used the corresponding SMB extract as antigen. None of the serums was infected simultaneously by two or more DENV serotypes. The serums of Group 1 scored as positive for IgG to DENV in an indirect ELISA performed on an additional sample collected during the convalescent phase of the disease (day 15 or later). For the serums of Group 2, all the tests were negative. The methodologies for the collection of the serum samples, the collection of the associated clinical data, and the characterization of the serums have been described previously in detail [65]. Data on the primary or secondary nature of the infection were not available. We obtained informed consent from the patients for the use of the Group 2 serum samples in a previous clinical study, as described [65]. The constitution of the above human serum collections (Group 1 and Group 2) and their use for the present study were approved by the Institutional Review Board of Institut Pasteur and a regional ethical committee (Comité de Protection des Personnes, Ile-de-France 1).

### MAC-ELISA

The MAC-ELISAs of the present study were performed in 96-well flat-bottom microtiter plates with a volume of 100 μL/well. The plates were sensitized with antibodies to human IgMs as follows. A goat antibody to human IgMs (1.0 μg/mL in PBS) was loaded in the wells of the plate. The plate was incubated overnight at 4°C for the reaction of adsorption. The wells were washed with buffer A (three times), blocked with buffer B for 1 h at 37°C, and then washed as above. The serums and recombinant antigens were diluted in buffer C. The serums were diluted 100-fold, a dilution at which they nearly saturate the IgM binding sites in the sensitized wells [66]. Wells were loaded with the diluted serums or with buffer C as a blank sample, and the plate was incubated for 1 h at 37°C for the reaction of antibody capture. The wells were washed as above and then loaded with the solution of recombinant antigen. The plate was incubated for 1 h at 37°C for the binding reaction. The wells were washed as above and the bound antigen, (H6-ED3-PhoA)_2_, was detected by addition of 5 mM pNPP in buffer D and measurement of *A*_405nm_ after 3 h at 25°C. Each experimental data point was performed at least in duplicate and the corresponding signals were averaged. The serum specific signal was obtained by subtracting the signal of the blank sample from the signal of the serum sample.

### Analysis of the experimental data

The curve fits were performed with Kaleidagraph (Synergy Software), which gives Pearson’s coefficient of correlation, *R*_P_. The mean values, standard deviations (SD) and standard errors (SE) were calculated with the same program. The results of the MAC-ELISAs were analyzed through ROC curves, which are equivalent to Wilcoxon statistics [67,68]. A ROC curve relates the False Positive Fraction (FPF = 1 - specificity) to the True Positive Fraction (sensitivity) of a test when the threshold varies. The area under the ROC curve (AUC) is an unbiased measure of the test accuracy and the difference AUC - 0.5 is the discrimination power or more simply discrimination of the test. An AUC value of 1.0 represents a perfect test whereas a value of 0.5 represents a worthless test. A rough guide for classifying the accuracy of a test has been proposed: 0.90-1.0 = excellent, 0.80-0.90 = good, 0.70-0.80 = fair, 0.60-0.70 = poor, 0.50-0.60 = fail. Semi-parametric ROC curves were computed with the LABROC4 program [69] as implemented in the Web based calculator JLABROC4 [70].

## Results

### Rationale

To analyze the serotype specificities and cross-reactivities of IgMs directed against the ED3 domains of the dengue viruses, we used a collection of 179 well-characterized human serums (see Methods). These serums belonged to two groups. The first group included four categories of infected serums, i.e. 18 serums infected by DENV1, 24 by DENV2, 18 by DENV3 and 30 by DENV4 respectively, for a total of 90 DENV-infected serums. Samples of these serums reacted positively in a MAC-ELISA that used a whole homotypic DENV antigen. The second group contained 89 DENV-uninfected serums. In a first step, we assayed each of the 179 serums in duplicate in four MAC-ELISAs that used four (H6-ED3-PhoA)_2_ dimers as antigens. The ED3 domains of these four reagents came from each of the four serotypes of DENV and we will designate these reagents as R1 to R4 in the following. The mean signal values and associated SD and SE of the MAC-ELISAs performed with R1 to R4 on the five categories of serums above are reported in Table 3. In a second step, we combined the serums in different sets that we considered as positive (+) or negative (−) for the hypothesis under test and analyzed the results of the MAC-ELISAs with ROC curves. More specifically, we derived a unique parameter for each test from these analyses, the precision (AUC) or discrimination (AUC – 0.5), and compared them between tests.

**Table 3:** **Mean signals of MAC-ELISAs performed with (H6-ED3-PhoA)**_**2 **_**dimers on five categories of serums**

**Serum**	**#**	**R1**	**R2**	**R3**	**R4**
DENV1	18	1.3	0.7	0.3	0.06
		± 1.4 (0.3)	± 1.1 (0.2)	± 0.5 (0.1)	± 0.04 (0.01)
DENV2	24	0.07	0.5	0.11	0.038
		± 0.09 (0.02)	± 0.5 (0.1)	± 0.14 (0.03)	± 0.020 (0.004)
DENV3	18	0.08	0.19	0.23	0.04
		± 0.11 (0.03)	± 0.20 (0.05)	± 0.21 (0.05)	± 0.09 (0.02)
DENV4	30	0.06	0.08	0.061	0.11
		± 0.16 (0.03)	± 0.09 (0.02)	± 0.049 (0.009)	± 0.22 (0.04)
None	89	0.008	0.058	0.077	0.044
		± 0.021 (0.002)	± 0.051 (0.005)	± 0.075 (0.008)	± 0.023 (0.002)

R1 to R4, (H6-ED3-PhoA)_2_ dimers of serotypes DENV1 to DENV4, respectively. The first and second columns give the serotype of the infecting DENV and the number of samples for each category of serums. None, DENV-uninfected serums. The other entries give the mean A_405nm_ value and associated SD and SE (in parentheses) for MAC-ELISAs performed on the serums of the first column with reagents R1 to R4.

### Discrimination between DENV-infected and -uninfected serums

In a first step, we analyzed the capacity of the four reagents, R1 to R4, to discriminate between IgMs of patients infected by a DENV serotype, either homotypic or heterotypic to the reagent, and IgMs of DENV-uninfected patients. The serums of the infected patients were considered as positive and the serums of the uninfected patients as negative. The discrimination between the IgMs of the infected serums and the IgMs of the uninfected serums was the highest when the reagent and infecting DENV were homotypic. These homotypic discriminations were excellent for DENV1, good for DENV2, fair for DENV3 and poor for DENV4 (diagonal of Table 4). Therefore, the serums of patients infected by any of the DENV viruses formed IgMs against the homotypic ED3 domain and could be recognized by the homotypic reagent.

**Table 4:** **Capacity of the (H6-ED3-PhoA)**_**2 **_**reagents to discriminate between DENV-infected and DENV-uninfected serums in a MAC-ELISA**

**(+) Serums**	**R1**	**R2**	**R3**	**R4**
DENV1	0.94 ± 0.05	0.83 ± 0.06	0.64 ± 0.08	0.59 ± 0.08
DENV2	0.90 ± 0.03	0.88 ± 0.05	0.57 ± 0.06	0.42 ± 0.07
DENV3	0.82 ± 0.06	0.75 ± 0.07	0.77 ± 0.07	0.18 ± 0.08
DENV4	0.73 ± 0.05	0.55 ± 0.06	0.45 ± 0.06	0.61 ± 0.07
DENV1-4	0.83 ± 0.03	0.73 ± 0.04	0.59 ± 0.04	0.47 ± 0.04

R1 to R4, (H6-ED3-PhoA)_2_ dimers of serotypes DENV1 to DENV4, respectively. The first column gives the serotype of the infecting DENV for the (+) serums. DENV1-4, the serotype of the infecting virus could be any one of DENV1 to DENV4. The DENV-uninfected serums were always taken as (−) serums. Each entry gives the accuracy and associated standard error of a MAC-ELISA that used the R_i_ reagent (i = 1, …, 4) as an antigen and was evaluated on the (+) and (−) serums.

Reagent R1 discriminated between the IgMs of DENV2-, DENV3- or DENV4-infected serums and IgMs of DENV-uninfected serums with excellent (DENV2), good (DENV3) or fair (DENV4) accuracies (column 1 of Table 4). Therefore, the IgMs of serums infected by DENV2, DENV3 or DENV4 cross-reacted with the ED3 domain from DENV1 (ED3.DENV1). By a similar reasoning, we concluded that the IgMs of serums infected by DENV1 or DENV3 cross-reacted with ED3.DENV2 but not the IgMs of serums infected by DENV4 (column 2 of Table 4). The IgMs of serums infected by heterotypic DENV did not cross-react significantly with ED3.DENV3 and ED3.DENV4 (columns 3 and 4 of Table 4). We concluded that there exist inherent cross-reactivities between the ED3 domain of a given DENV serotype and the human IgMs directed against DENV heterotypes.

### Relation between reagent serotype and test accuracy

Table 4 enabled us to compare the accuracies of MAC-ELISAs performed with R1 to R4 when run on the same two sets of serums, infected and uninfected. For example, the discrimination between the DENV1-infected serums and the DENV-uninfected serums was excellent for R1, good for R2, poor for R3 and nil for R4 (row 1 of Table 4). Similarly, the discrimination between the DENV2-infected and DENV-uninfected serums was excellent for R1 and R2 and nil for R3 and R4 (row 2 of Table 4). The discrimination between DENV3-infected and DENV-uninfected serums was good for R1, fair for R2 and R3, and nil for R4 (row 3 of Table 4). The discrimination between DENV4-infected and DENV-uninfected serums was fair for R1, nil for R2 and R3 and poor for R4 (row 4 of Table 4). The discrimination between DENV_j_-infected serums and DENV-uninfected serums by the R_i_ reagent was not linearly correlated with the number n_i,j_ of residue changes between ED3.DENV_i_ and ED3.DENV_j_ (i ≠ j) (*R*_P_ = 0.54). These comparisons suggested that the levels of discrimination depended on the specific couple of DENV serotypes and not on the sequence differences between the heterotypic ED3 domains. Whether and how the levels of discrimination of the same two sets of (+) and (−) serums by the different reagents might be related to the strengths of the IgMs reactivities is considered in the Discussion section.

The homotypic R_i_ reagent and some heterotypic R_j_ reagents could discriminate between DENV_i_-infected serums and DENV-uninfected serums (i ≠ j). How did these homotypic and heterotypic discriminations compare? The discriminations by R1 were higher than or equal to those by the homotypic reagents for any infecting serotype, DENV2, DENV3 or DENV4 (compare the numbers in column 1 and in the diagonal of Table 4). The discriminations by R2 were roughly equal to those by the homotypic reagents in the same conditions. In contrast, R3 and R4 did not recognize serums that were infected by heterotypic DENVs. These results were not due to the serums and their IgMs since the serums were identical for the four reagents. They were necessarily due to differences in the antigenic properties of the ED3 domains of the four DENV serotypes since the (H6-ED3-PhoA)_2_ constructions were exactly identical except for this domain.

### Discriminations between DENV serotypes

In a second step, we analyzed the accuracy with which a given R_i_ reagent discriminated between DENV_i_- and DENV_j_-infected serums, with i ≠ j (Table 5). The R1 reagent discriminated between the DENV1-infected serums and the DENV2-, DENV3- or DENV4- infected serums with good accuracies (column 1 of Table 5), despite important cross-reactions between ED3.DENV1 and the IgMs of serums infected by heterotypic DENVs (column 1 of Table 4). The R2 reagent discriminated between DENV2-infected serums and either DENV3- or DENV4-infected serums with fair to good accuracies but it did not discriminate between DENV2- and DENV1-infected serums (column 2 of Table 5), in accordance with the cross-reaction pattern (column 2 of Table 4). The R3 reagent discriminated between DENV3-infected serums and either DENV1- or DENV2-infected serums with poor to no accuracies (column 3 of Table 5), despite weak cross-reactions between ED3.DENV3 and the IgMs of DENV1- or DENV2-infected serums (column 3 of Table 4). In contrast, R3 discriminated between DENV3- and DENV4-infected serums with a good accuracy, in accordance with the absence of cross-reactions between ED3.DENV3 and the IgMs of DENV4-infected serums. The R4 reagent did not discriminate between DENV4- and DENV1-infected serums (column 4 of Table 5), despite the absence of cross-reactions between ED3.DENV4 and the IgMs of DENV1-infected serums (column 4 of Table 4). R4 discriminated between DENV4-infected serums and either DENV2- or DENV3-infected serums with good accuracies, in accordance with the absence of cross-reactions between ED3.DENV4 and the IgMs of such serums. A more detailed analysis of the data in Tables 4 and 5 showed that the discrimination by a given R_i_ reagent between homotypic DENV_i_- and heterotypic DENV_j_-infected serums was not correlated with the discriminations by R_i_ between DENV_j_ infected serums and DENV-uninfected serums (not shown). Otherwise stated, the R_i_ reagent may not discriminate between the DENV_i_- and DENV_j_-infected serums, even if the IgMs of the DENV_j_-infected serums do not cross-react with ED3.DENV_i_. Conversely, the R_i_ reagent may discriminate between the DENV_i_- and DENV_j_ serums even though the IgMs of the DENV_j_-infected serum cross-react with ED3.DENV_i_. Moreover, the discrimination by the R_i_ reagent between DENV_i_- and DENV_j_-infected serums was not correlated with the number of residue changes between ED3.DENV_i_ and ED3.DENV_j_ (not shown). Thus, sequence differences could not predict the discrimination between two serotypes by a given reagent.

**Table 5:** **Capacity of the (H6-ED3-PhoA)**_**2 **_**reagents to discriminate between serums infected by different DENV serotypes in a MAC-ELISA**

**(−) Serums**	**R1**	**R2**	**R3**	**R4**
DENV1	na	0.52 ± 0.09	0.55 ± 0.10	0.57 ± 0.08
DENV2	0.78 ± 0.08	na	0.68 ± 0.08	0.70 ± 0.07
DENV3	0.84 ± 0.07	0.72 ± 0.08	na	0.80 ± 0.07
DENV4	0.87 ± 0.06	0.85 ± 0.05	0.81 ± 0.07	na
Other DENV	0.84 ± 0.06	0.72 ± 0.06	0.70 ± 0.06	0.69 ± 0.06
All others	0.90 ± 0.05	0.81 ± 0.04	0.73 ± 0.06	0.66 ± 0.06

R1 to R4, (H6-ED3-PhoA)_2_ dimers of serotypes DENV1 to DENV4, respectively; na, non applicable. The serotype of the infecting DENV for the (+) serums was always identical with the serotype of the reagent. The first column gives the serotype of the infecting DENV for the (−) serums. Other DENV, all DENV serotypes except the serotype of the reagent. All others, all serums (infected and uninfected) except those infected by the same DENV serotype as the reagent. Each entry gives the accuracy and associated standard error of a MAC-ELISA that used the R_i_ reagent (i = 1, …, 4) as an antigen and was evaluated on the (+) and (−) serums. Note that the expressions (+) serums and (−) serums refer to the discrimination under statistical analysis and not to the infected or uninfected character of the serums.

### Additional discriminations

For each R_i_ reagent, we calculated its discrimination between serums infected by any of the four DENV serotypes (DENV-infected serums) and DENV-uninfected serums, i.e. the set of positive serums was constituted by all the DENV_j_-infected serums with j = 1, …, 4. This discrimination was good for R1, fair for R2 and nil for R3 and R4 (Table 4, row 5). The discrimination between the DENV-infected and DENV-uninfected serums by R_i_ was strongly negatively correlated with the number of residue changes between the consensus ED3 domain and the ED3.DENV_i_ domain (*R*_P_ = 0.98; compare rows 5 of Tables 2 and 4). Thus, ED3.DENV1 behaved like a consensus ED3 domain.

For each reagent R_i_ (i = 1, …, 4), we calculated its discrimination between serums infected by the homotypic DENV_i_ and serums infected by any of the three heterotypic DENVs, i.e. the set of negative serums was constituted by all the DENV_j_-infected serums with j ≠ i. This discrimination was good for R1 and fair for R2, R3 and R4 (Table 5, row 5). We calculated its discrimination between serums infected by the homotypic DENV_i_ virus and serums uninfected by any DENV or infected by any of the three heterotypic DENV_j_ (j ≠ i). This discrimination was excellent for R1, good for R2, fair for R3 and poor for R4 (Table 5, row 6). Thus, each R_i_ reagent could recognize the homotypic DENV_i_-infected serums with at least some accuracy.

## Discussion

### MAC-ELISA using (H6-ED3-PhoA)_2_ dimers

The ability to analyze the specificities and cross-reactivities of human IgMs towards the different serotypes of the ED3 domain depends on the existence and use of a reliable test for the interaction between IgMs and ED3 domains. Here, we used a MAC-ELISA with (H6-ED3-PhoA)_2_ dimers as antigens. The use of such antigens raised two questions: was the ED3 domain correctly folded in these dimers and was it accessible to antibodies? The ED3 domain has one disulfide bond and the PhoA monomer has two disulfide bonds. Previously, we have shown that the isolated ED3 domain can be produced in a correctly folded state in the periplasmic space of *E. coli,* where the formation of the disulfide bonds is efficiently catalyzed [71]. In particular, site-directed mutagenesis experiments and the crystal structures of the complexes between the ED3 domains from the four serotypes of DENV and the scFv fragment of the broadly neutralizing monoclonal antibody mAb4E11 have shown that the epitope of mAb4E11 is discontinuous, conformational, and included within the ED3 domain [29,72]. PhoA is enzymically active only as a dimer. We have shown that the H6-ED3-PhoA hybrids have both their ED3 and PhoA portions correctly folded and active when they are produced in the *E. coli* periplasm. This result was obtained by measuring the specific activity of the hybrids for the dephosphorylation of pNPP in vitro and assaying their binding to immobilized mAb4E11 in an indirect ELISA, revealed with their intrinsic phosphatase activity [66].

In the H6-ED3-PhoA hybrids, the C-terminal residue of ED3 (residue 400 of the E protein) is linked to residue Val7 of the mature PhoA through a flexible linker tripeptide Thr-Ser-Gly [66]. The (H6-ED3-PhoA)_2_ dimers recognize both mAb4E11, as recalled above, and cell receptors [41,73]. Therefore, the ED3 domain should be at least as accessible in the (H6-ED3-PhoA)_2_ dimers as in the full and infectious DENV virus, where it interacts with the other domains of the E protein and its C-terminal residue is linked to the transmembrane region of the E protein and faces the lipid membrane [74].

Thus, the (H6-ED3-PhoA)_2_ dimers can be directly produced in a soluble, correctly folded, multimeric state in the periplasmic space of *E. coli.* They can be produced and purified in a homogeneous state from periplasmic extracts. They constitute self-sufficient reagents since the ED3 antigen and PhoA reporter enzyme are covalently linked within the same molecule. MAC-ELISA based on such dimers involve a low number of steps or manipulations. The antigen is dimeric and therefore can bind its target through an avidity phenomenon. Such dimers have already been used to detect weak interactions between the ED3 domains of various flaviviruses and either cell receptors or human IgMs [41,71,73].

### Analysis of MAC-ELISAs with ROC curves

The analysis of a test with a ROC plot gives a measure of its capacity to distinguish between two alternatives, the positive and negative cases. A ROC plot gives the accuracy (AUC) value of a test independently of the event frequencies in the test samples and of the decision criterion, i.e. threshold value. To measure the accuracies of the MAC-ELISAs that used the (H6-ED3-PhoA)_2_ dimers as antigens, it was necessary to constitute collections of well-characterized human serum samples, i.e. serums whose DENV-infected or uninfected status was reliably established, whose infecting DENV serotype was known, and which contained significant amounts of IgMs against the infecting DENV serotype. These characterizations are described briefly in the Methods section and in more detail elsewhere [65]. They depended themselves on specific tests and thresholds. However, because they were based on sensitive, well-established, standardized, redundant methods that differed from that under analysis, we considered the classifications of the human serums in our collection as absolute.

An AUC value of *x* means that a randomly selected serum with the properties of the (+) group has a test value larger than that of a randomly chosen serum with the properties of the (−) group 100*x*% of the time [67]. Therefore, if a MAC-ELISA with reagent (H6-ED3-PhoA)_2_ can discriminate between the serums of the (+) and (−) groups, this discrimination implies that the interaction with the ED3 domain is “stronger” for the IgMs of the (+) group than for the IgMs of the (−) group, where the word “stronger” may include both concentration and avidity components.

### Serotype cross-reactivities of human IgMs

Our results confirmed that the serums of human patients infected by DENV contain IgMs directed against the ED3 domain [46-48]. They showed that the IgMs of human serums infected by a given DENV_i_ serotype cross-reacted with the ED3 domains from other DENV_j_ serotypes (i ≠ j). Therefore, they extended similar observations, previously made with whole-virus antigens, to the small ED3 domain [15-19]. They also extended similar observations, previously made for IgGs, to IgMs [75]. The exact patterns of cross-reactivities depended on the infecting DENV serotype.

The ED3.DENV_i_ domains could discriminate between the IgMs of serums that were infected by the homotypic DENV_i_ virus and the IgMs of serums that were uninfected by DENV, in our MAC-ELISA assays. The levels of discrimination varied with the serotype, in the decreasing order DENV1 ≥ DENV2 ≥ DENV3 > DENV4 (diagonal of Table 4). This conclusion on IgMs is reminiscent of published data on IgGs: (i) Several studies have shown that many mouse monoclonal antibodies that are specific for the DENV complex of flaviviruses, have affinities for the four serotypes of the ED3 domain and neutralization potencies of the four serotypes of DENV in the same decreasing order as above [25,29,76]. (ii) The content of human serums in IgGs against DENV after a primary infection is higher during an infection by DENV1 and lower during infections with DENV2 and DENV3 when assayed by an indirect ELISA with recombinant ED3 domains as antigens [6]. (iii) In tetravalent strategies of immunization against dengue simultaneously with attenuated strains, or E proteins, or ED3 domains of the four DENV serotypes, lower levels of neutralizing antibodies are induced against DENV3 and especially DENV4 in comparison with DENV1 and DENV2 in mice and humans [59,62,77,78]. These comparisons between our results on IgMs and published data on IgGs suggest that the AUC value might be somehow related to the “level” or “strength” of the interaction between the serum IgMs and ED3 domain under assay, in our specific experimental conditions. If such a relation was valid, we could conclude that the serum IgMs reacted the strongest with ED3.DENV1 and the weakest with ED3.DENV4, whatever the infecting serotype (rows of Table 4).

The R1 reagent gave the highest discrimination between serums infected by any one of the DENV serotypes and uninfected serums. In particular, R1 gave a discrimination equal to or higher than that of the homotypic reagent, between serums infected by a given DENV serotype and uninfected serums (Table 4). These results showed that ED3.DENV1 behaved like a consensus or universal ED3 domain for the detection of IgMs directed against any DENV serotype. They were reminiscent of published data showing that, in human secondary infections, the serum titer in IgGs against ED3.DENV1 is higher than those against the three other ED3 serotypes, regardless of the infecting DENV serotype [6].

### Serotype specificities of human IgMs

We showed that some R_i_ reagents could discriminate between human IgMs directed against the homotypic DENV_i_ and IgMs against heterotypic DENV_j_ viruses (j ≠ i). The levels of discrimination by R_i_ between IgMs directed against the two different DENV serotypes were not correlated with the levels of discrimination by R_i_ between DENV_j_ infected serums and DENV-uninfected serums, i.e. with the profiles of cross-reactivities. They were not correlated with the numbers of residue changes between ED3 domains. Noteworthily, R1 could discriminate between IgMs against DENV1 and IgMs against DENV2, DENV3 or DENV4, whereas R2, R3 and R4 could not discriminate between IgMs against the homotypic DENV and IgMs against DENV1 (Table 5). Any ED3 domain interacted more strongly with the IgMs against the homotypic DENV than with the IgMs against heterotypic DENVs, with one exception. Each ED3 domain interacted as strongly with the IgMs against DENV1 as with the IgMs against the homotypic DENV (row 1 of Table 5). Fine structural properties might explain these discriminations between serotypes. The R_i_ reagents had also significant levels of discrimination towards IgMs directed against the heterotypic DENV_j_ viruses (j ≠ i), taken as a whole (line 5 of Table 5), although not high enough for diagnostic tests. Data on the discrimination between DENV serotypes by ED3 domains in a MAC-ELISA had not been reported previously to our knowledge.

## Conclusions

Many properties of serotype specificity and cross-reactivity that have been reported for IgGs against the ED3 domain of DENV, appear to pre-exist in IgMs. These properties could thus be passed from IgMs to IgGs during the maturation of the immune response.

Our study describes tools, the (H6-ED3-PhoA)_2_ dimers, and a statistical method that can be used to characterize the early immune response against different serotypes or strains of pathogenic flaviviruses, in particular after a vaccination and challenge. Its results for the four DENV serotypes should help better understand the early immune response during infections by these viruses. These results could be useful for the interpretation of MAC-ELISA assays that are used in the diagnosis of dengue, and for the fine engineering of the ED3 domains to obtain better diagnostic reagents and vaccines.

## Competing interests

Patent applications that include the use of the (H6-ED3-PhoA)_2_ dimers as antigens in MAC-ELISA have been filed by Institut Pasteur and CNRS. Should these institutions receive revenues as a result of licensing, the authors are entitled to receive payments through the respective Inventor’s Rewards Schemes. Our funding sources had no role in the conduct of the research or the preparation of the article.

## Authors’ contributions

NZ prepared the (H6-ED3-PhoA)_2_ hybrids. LB carried out the immunoassays. PD constituted the collection of human serums and supervised the immunoassays. HB coordinated the study and was responsible for the final versions of the data analysis and paper. All authors participated in the analysis of the data. All authors read and approved the final manuscript.

## Pre-publication history

The pre-publication history for this paper can be accessed here:

http://www.biomedcentral.com/1471-2334/13/302/prepub
